# Benefits of a Low-Cost Walking Device in Children with Cerebral Palsy: A Qualitative Study

**DOI:** 10.3390/ijerph18062808

**Published:** 2021-03-10

**Authors:** Isabel Rodríguez-Costa, Irene De la Cruz-López, Ignacio Fernández-Zárate, Saturnino Maldonado-Bascón, Sergio Lafuente-Arroyo, Susana Nunez-Nagy

**Affiliations:** 1 Department of Physical Therapy, University of Alcalá, 28807 Alcalá de Henares, Spain; irene.cruz@edu.uah.es (I.D.l.C.-L.); ignacio.fernandezz@edu.uah.es (I.F.-Z.); susana.nunez@uah.es (S.N.-N.); 2 Department of Signal Theory and Communications, University of Alcalá, 28807 Alcalá de Henares, Spain; saturnino.maldonado@uah.es (S.M.-B.); sergio.lafuente@uah.es (S.L.-A.)

**Keywords:** low-cost technology, self-help device, cerebral palsy, qualitative research

## Abstract

Children with Cerebral Palsy (CP) participate less regularly in physical and social activities. Support walkers allow mobility for infants who need aid. The aim of this study is to explore the benefits of a low-cost walking device in children with CP. A qualitative study using semi-structured, face-to-face interviews was conducted. Eight participants (two parents, two educational professionals, and four physical therapists) who live or work with children with CP that use a low-cost walking device were questioned to examine the benefits of the practice. Thematic analysis denoted three key factors about the benefits: emotional welfare, physical wellbeing, and social enjoyment. To conclude, the use of a support walker in children with CP makes them feel happier, improves their self-confidence and autonomy, and promotes participation.

## 1. Introduction

Cerebral palsy (CP) is described as a collection of persistent disorders of movement and posture, resulting in activity and participation limitations [1]. Infants with CP are involved less frequently in physical and public activities, and in less varied activities than children without CP [2]. Infants with CP have reduced levels of participation due to the categorization level of the Gross Motor Classification System [3] and complicated degrees of sedentary behaviors [4], which have health consequences [5,6]. 

Walking capability is one of the intervention objectives in children with CP. To achieve functional walking, infants require appropriate muscle strength, nerve innervation, good endurance, and less fatigue [7]. Walking is more effortful for children with CP than it is for their non-disabled peers due to weakness, lack of coordination between muscle groups, flexed posture, poor balance, and altered muscle tone [8]. Walking devices improve performance, stability, and posture. Posterior walkers (PW) were created in the 1980s to facilitate an upright walking stance [9].

For more active CP children, support walkers are a division of assistive devices that may enable non-ambulant infants with disabilities to walk on their own and promote improvements in mobility, autonomy, participation, and social function [10]. Support walkers are described as instruments that allow mobility for infants who need more aid than that supplied by handheld walkers, including some level of trunk, pelvic, and/or head support [11].

Support walkers may give non-ambulant infants the opportunity to walk independently in a challenging context, such as at school. Variations in the level of participation in school activities among children with or without CP have been evidenced [12]. These observations highlight the need to promote an intervention approach aimed at overcoming learning and environmental barriers to support inclusive education [13]. 

In this framework, an initiative named “*Padrino Tecnológico*”, from Alcalá University, has emerged to design and develop low-cost support walkers for the community [14]. Every year, this program donates around ten devices across the 37 beneficiary organizations. To date, previous studies of the benefits of support walkers for children with disabilities have been conducted using a quantitative design [10]. This approach limits the collection of information from participants, as they are restricted in expressing their thoughts and opinions in a more detailed way. Due to the complexity of the topic, qualitative research is required to explore this area of concern. To the best of our knowledge, this is the first study to examine these factors. The aim of this study was to describe the experience of utilizing a walker developed by “*Padrino Tecnológico*”—a low-cost walking device for daily life.

## 2. Materials and Methods

### 2.1. Study Design

A qualitative research study was developed. Qualitative investigations are helpful for understanding the impressions, values, and motivations of the observed phenomena [15]. In this paper, the event under review was the benefits of using a low-cost walking device for children with CP in school, elaborating a phenomenological study. The study was based on semi-structured, face-to-face interviews to explore this phenomenon. Various participants (students, teachers, physical therapists, and families), contexts, places, and moments connected to the event were studied [16]. 

This study was carried out in an academic context, in three schools of the Madrid Community, Spain. Ethical approval was gained from the Office of the Vice President for Research and Transfer of Alcalá University and CPPE Juan XXIII (number 204, date 14 November 2013).

### 2.2. Participants

Participants in this research were selected from parents, physical therapists, and educational professionals of children who used the support walker donated by “*Padrino Tecnológico*”. Subjects who could provide knowledge regarding the event under study were included. The inclusion criteria for the participants of this project were:

(a) Family context: parents of children with a diagnosis of CP, between the ages of 3 and 18, who have used the support walker at least for one year.
(b) Educational and therapy context: teachers and physical therapists of children with a diagnosis of CP, who have used the support walker in the school where they work for at least one year.

All enlisted participants gave informed consent prior to participating in the study. The study took place in three different schools of the Madrid Community, Spain. Finally, two mothers, four physical therapists, and two educational professionals were included. Sociodemographic data of infants whose parents participated were added to contextualize the study (Table 1).

### 2.3. Procedure

Information selection consisted of two stages. In the first stage, the researchers explained the purpose of the study to the school principal. Then, there was a second meeting with physical therapists and educational professionals to detail the project’s design, and questions about the study were answered. Physical therapists made the connection with the families. Participants who agreed to take part in the study were given a date for the face-to-face interview, which took place in a comfortable room of the three schools. 

In the second stage, in-depth interviews were conducted. The face-to-face, in-depth interviews concerned six phases: thematizing, designing, interviewing, transcribing, analyzing, and verifying [17]. The interviews were administrated by a researcher who had not been involved in any treatment. An inquiry manual was used to explore relevant spheres of the project to tackle the aim of the study [16]. The query was guided in an open way to avoid subjects’ direct answers (Table A1). Face-to-face interviews were conducted in Spanish, as chosen for all participants. Interviews were audio-recorded for successive transcription. Data saturation was reached when no new concept was communicated and there was a recurrence of ideas among the subjects [18]. 

### 2.4. Data Analysis

Audio recordings from the in-depth interviews were transcribed verbatim for each meeting in its entirety and were analyzed using Atlas.ti software (Version 5.4, Scientific Software Development, Berlin, Germany). A thematic and inductive analysis was carried out by two researchers (I.R.-C. and I.C.-L). Thematic analysis tries to identify the most descriptive content to obtain codes, and then decrease and recognize the most common categories. In this way, clusters of codes (categories) were formed into content that allowed the appearance of themes explaining the study participants’ experience of the benefits of using a support walker [16]. 

### 2.5. Credibility and Qualitative Rigor

The standards for guaranteeing trustworthiness by Guba and Lincoln were applied [19]. Triangulation in data analysis was guaranteed by procuring two researchers to independently read and understand the verbatim transcripts. Quotations employed by participants were incorporated, where possible, in the creation of codes and themes, in order to increase credibility [20].

## 3. Results

Eight participants (two parents, one teacher, one specialist teacher, and four physical therapists) who live or work with children with CP who use a low-cost walking device participated in this study. Interviews ran for about 45–60 min each. Table 1 shows the sociodemographic data of the participants. The mean age of the children was 8.75 years (SD ± 5.5), the mean duration of school attendance was 5.75 years (SD ± 4.5), and the mean number of years using the walker was 2.5 years (SD ± 1). Three themes emerged from the material analyzed: (a) emotional welfare, (b) physical wellbeing, and (c) social enjoyment. In the following sections, some of the participants’ narratives—obtained from the interviews regarding the three emerging themes—are detailed [21]. 

### 3.1. Theme 1—Emotional Welfare

Regarding the emotional aspect, the interviewees indicated the importance of improvements in the general mood of the child. Children who used the walker with weight-bearing loads were more cheerful and happy, and this aid allowed them to socialize more with their environment.

*“His way of being changed a lot, he began to be more cheerful and interacted with other classmates…”* (P.A)

The most cited codes of this theme (18 and 13 citations) were “joy” and “happiness”. Both are closely related and complement each other. All participants highlighted that the children’s moods had improved significantly with the use of the walker.

*“When he walked I saw him quite happy, he was happy …”* (F.A)

*“She was delighted, she really likes to walk, explore the environment …”* (P.R)

*“She was happy, she likes walking a lot.”* (P.R)

Another relevant aspect, related to state of mind, is that some of the interviewees had witnessed a significant decrease in the frequency of the child’s crying:

*“He was less angry; he didn’t cry so much…”* (P.A)

In respect of self-esteem, the interviewees highlighted changes related to the use of the walker developed by “*Padrino Tecnológico*”.

*“We were looking above the child’s autonomy, self-esteem, trunk control, and balance. All these aims have been achieved, so very well.”* (P.L)

Family members also highlighted how exciting it was to be able to see their son standing and walking—milestones according to his age—in contrast to the previous moments when they only saw him moving on the ground.

*“It was fascinating for me that he walked, since before he was only on the ground.”* (F.A)

### 3.2. Theme 2—Physical Wellbeing:

In this theme, all the codes related to benefits caused in structure and function were grouped, including improvement of intestinal rhythm, tolerance to effort, strength gain, standing, autonomous gait, improvement of balance, and prevention of deformities.

Regarding the improvement of intestinal rhythm, relatives pointed out that the walker has a favorable effect, as it is weight-bearing and allows standing and walking, which were not otherwise possible.

*“…The more he moves the better she goes to the bathroom. Also, my daughter is constipated and that shows a lot.”* (F.R)

Concerning effort tolerance, all respondents agreed with the fact that walker training facilitated improvements in the physical resistance of the beneficiaries.

*“Yes, it is true that now they get tired later than when they started using it.”* (P.L)

*“At the beginning, above all, he got tired a lot, but he got used to it.”* (F.A)

Linked to the increase in the fatigue threshold, the professionals highlighted an advantage in strength.

*“Also that they gain strength in their legs to go up ramps alone, which in this latter have already improved a lot…”* (P.L)

In this thematic unit, the most cited code by the participants was the improvement of balance (with 19 citations). Physiotherapists of the centers highlighted improvements in the reactions of straightening and balance, as well as the good control of the trunk and pelvis that the device provides to these children.

*“Furthermore, it’s a walker that controls you [of the children] very well at the level of the trunk, pelvis, lower limbs …”* (P.R)

*“Yes, it is quite visible that he has more trunk control; they do not go forward or to the sides …”* (P.L)

*“It is a good tool to work the gait when the girl was growing up, the reactions of straightening and balance.”* (P.R)

Linked to balance, they also highlighted (with 18 citations) the ability for standing and autonomous walking that this device provides to the children, which, due to their motor characteristics, would not be possible another way.

*“The first thing, it allows walking or reproducing the gait gesture in children who could not.”* (P.A)

*“It reproduces a suitable gait pattern; it facilitates him walking much easier, the heel attack…”* (P.L)

*“Before using the Walker, he did not get up from the ground, and now he walks with little help …”* (F.A)

Interviewees highlighted that this aspect had a relevant impact on reducing falls, as well as promoting a feeling of security while walking that facilitates their integration.

*“No, they haven’t had a fall currently.”* (P.L)

*“You were sure that it was not going to fall.”* (P.A)

All these characteristics of the walker developed by “*Padrino Tecnológico*” also make it possible for it to be a technical aid, contributing to the prevention of injuries.

*“It was also an objective the prevention of deformities, hip dislocations …”* (P.R)

### 3.3. Theme 3—Social Enjoyment:

In this third theme of results, several benefits were found and collected by the participants, such as increased sociability, improved displacements, increased hands use, interaction with the environment, increased autonomy, and no economic costs.

On the one hand, the social isolation that is usually related to people with disabilities was reduced because, after using the walker developed by “*Padrino Tecnológico*”, these children felt more confident and safe, meaning they were more sociable.

*“We tried putting him in the walker, and as he looked well placed, his attitude changed. Now he socializes much better.”* (F.A)

*“He was much more integrated with the rest, really. Before, he was isolated and didn’t want to know anything.”* (P.A)

On the other hand, participants improved their independence by moving from one place to another without help. This mobility facilitates their inclusion in educational and social activities which require displacements.

*“They are able to make displacements to the different activities that they have …”* (P.L)

Another code that emerged in this theme was the capability of using their hands. Children who used this walker were able to integrate their free hands, as this aid is different from other walking devices where upper limbs need to give support. This freedom improves their inclusion in activities with their peers.

*“Using their hands on the same level as everyone else.”* (P.L)

*“Having their hands free gives them many possibilities.”* (P.L)

Related to the availability of the hands, participants could change their interactions with their environment. This is a generous impulse for sensorimotor development.

*“In the classroom, he liked to explore the different corners.”* (P.R)

*“The perception of space from the standing helps them to understand spatial concepts and mathematics.”* (P.L)

One of the more notable codes of this theme was the increase in autonomy of the walker users (16 quotations). Hand freedom, better mobility for displacements, less fatigue, increases in strength, and improvements in self-esteem are key points to facilitate autonomy.

*“They also feel more autonomous because they walk alongside the other classmates.”* (P.L)

*“The autonomy that it allows, to be able to explore the environment autonomously.”* (P.R)

*“Also the great joy, the energy and the gain of autonomy, the possibility of walking by his side …”* (F.R)

Finally, participation in the “*Padrino Tecnológico*” project, in which support walkers are donated to the schools, has contributed to increasing the benefits of this aid without any economic cost for the families, as the walkers are accessible to them.

*“We focus more on the family finances since it was donated without any cost.”* (P.R)

**Figure 1:**
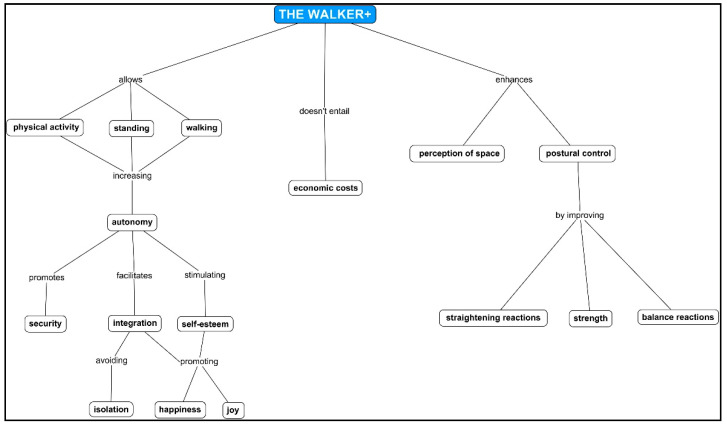
Walker benefits. Final network.

Figure 1 shows a unification of the benefits collected. Participants think that the walker makes it possible for the children to maintain an autonomous standing position and an autonomous gait, and that it facilitates the performance of physical activity. These features increase the autonomy of the individual, affecting their safety. Moreover, the autonomy provided by the walker facilitates the integration of the child into society, thus avoiding isolation and promoting the happiness of the user. It also increases the child’s level of self-esteem and promotes their joy. 

In addition, the walker developed by “*Padrino Tecnológico*” does not entail any economic cost for the child’s family, or for the school that acquires it. Finally, it improves the perception of space and the child’s postural control through straightening reactions, balance reactions, and strength. 

## 4. Discussion

The results of this research show the perceived benefits of the use of the support walker developed by “*Padrino Tecnológico*” in children with CP. Perspectives were collected from parents, physical therapists, and educational professionals in the context of educational schools. The findings from this study suggest several emotional, physical, and social benefits derived from the use of this device. 

In the present study, children were in the age range of 3–6 when they started using the support walker for 2.5 years (standard deviation [SD] +/−1). Physical therapists reported that the mean age of introduction to support walkers is 3.6 (1.6) years [10]. The youngest age of introduction, however, was reported as a mean of 2.9 (1.0) years (range 1–5 years) (10). Regarding the ideal age that a support walker could be introduced, 77 prescribers responded by giving a mean age of 2.5 (1.0) years (range 1–6 years), which again corresponds with the moment when the infants received the walker developed by “*Padrino Tecnológico*” [21]. Nevertheless, infants below the age of 3½ years old are habitually excluded from participating in studies analyzing the effectiveness of support walkers [22].

Although support walkers can be prescribed to infants with a substantial range of conditions, most children who use support walkers have cerebral palsy; a fact supported in our study [10].

The environments in which support walkers are most used are schools (100%), homes (72.2%), and in the community (38.9%). These data indicate the importance of the “*Padrino Tecnológico*” initiative, which donates walkers to schools [23].

In the literature review, there has been an extensive discussion about which walker—posterior or anterior—is better for children with disabilities. This walker was designed as a posterior walker, and data for other studies indicate the preference that users and parents have for a posterior walker [7]. This review suggests that the posterior walker can offer more stability than anterior devices [24]. 

Related to the physical benefits of the walker developed by “*Padrino Tecnlógico*”, effort tolerance was one of the main codes identified in this study. This is because other research referring to fatigue has been recognized by clinicians as a barrier to participation [25]. In this way, it has been demonstrated that a posterior walker, such as the walker developed by “*Padrino Tecnológico*”, promotes less oxygen cost [24]. Another physical benefit that emerged from the analysis was the improvement in muscle strength of the users’ lower limbs. In other studies, muscle strength is correlated with walking speed [26]. Another physical advantage is the ability to walk hands-free [27,28]. This is related to balance confidence that may also be a large burden on physical activity participation [29].

Concerning social benefits, this research harmonizes with the previous evidence about the increased willingness, independence, and quality of gait provided by a novel support device—a finding demonstrated by the children in this study [10,30,31]. As other studies suggest, social interests involve independence and peer meeting [10,27,28]. This support walker promotes participation, and it is well known that infants with cerebral palsy usually take part in less physical activity than their typically developing equals [10,28,29].

As the evidence suggests, participation in the community environment is essential for health, as it promotes an impression of feeling included, as well as opportunities for social contact and physical activity [17] that impact children’s moods and self-esteem. Community participation is vital for the wellbeing of young people. Data denote considerable inequalities in community participation—and health issues—for disabled young people when contrasted with non-disabled peers [32].

There were some limitations to this study. Firstly, given the relatively small sample size of eight, findings from the current study should be interpreted with caution and further consolidated in larger sample sizes. Secondly, it only reflects the perceived benefits of the use of a low-cost walking device in the Madrid Community. Next, the majority of the participants have a lot of experience working with children with disabilities (more than twenty years), and these data could be different in the general population. The preponderance of the subjects (parents) in this study were females (mothers); this is because mothers are the main caregivers for these children. For future research, children’s experiences and difficulties should be taken into account.

## 5. Conclusions 

These results may help to better understand the benefits of the use of the walker developed by “*Padrino Tecnológico*”. Low-cost support walkers could hold great hope for children with CP whose mobility options are limited. Emotional, physical, and social benefits are evident. Suggestions for future improvements and studies of use in activity and participation are provided.

## Figures and Tables

**Table 1:** Sociodemographic data of the participants.

Participants	Sociodemographic Data
Children	Participants: 4 (1 man, 3 women) Mean age (years): 8.75 (SD +/− 5.5)
	School attendance (years): 5.75 (SD +/− 4.5)
	Walker used (years): 2.5 (SD +/− 1)
Parents	Participants: 2 (2 women)
	Mean age (years): 42
Physical therapists	Participants: 4 (1 man/3 women)
	Mean age (years): 44.25 (SD +/− 6.3)
	Years of experience (years): 20.25 (SD +/− 5.3)
Education professionals	Participants: 2 (2 women)
	Mean age (years): 42.3 (SD +/− 13.4) Years of experience (years): 13.5 (SD +/− 16.3)

## Appendix A

**Table A1:** Questions squeme for face-to-face interviews.

Category	Examples of Questions
General data	Diagnosis of the users
	Time of use
	Adaptation
Mood and self-esteem	Improve of mood
	Improve of self-esteem
Breathing, bowel rhythm and feeding	Changes on these aspects
Pain and fatigue	Pain complaints
	Level of fatigue while using the walker
Functionality and autonomy	Activities that children can do with the walker
	Average distance travelled
	Spaces of use (house, school)
Balance	Improvements/changes
	Falls with the walker
